# Correlation of Fluorodeoxyglucose (FDG) PET Biomarkers (SUVmax, SUVpeak, TLG, and MTV) With Histopathological and Biochemical Parameters in FDG-Avid Lung Carcinoma

**DOI:** 10.7759/cureus.107331

**Published:** 2026-04-19

**Authors:** Anitha D, Priya S, Dhiren K Panda, Shishir Kumar

**Affiliations:** 1 Medicine, Vydehi Institute of Medical Sciences and Research Centre, Bangalore, IND; 2 Surgical Oncology, Vydehi Institute of Medical Sciences and Research Centre, Bangalore, IND; 3 Anatomy, Institute of Medical Sciences (IMS) &amp; SUM Hospital, Siksha 'O' Anusandhan University, Bhubaneswar, IND

**Keywords:** biochemical parameters, fdg pet, histopathology, lung carcinoma, mtv, suvmax, suvpeak, tlg

## Abstract

Background

Lung carcinoma remains a major cause of cancer-related mortality worldwide. Fluorodeoxyglucose positron emission tomography-computed tomography (FDG PET-CT) is a critical imaging modality in assessing tumor metabolic activity and burden. This study evaluates the correlation of FDG PET biomarkers, including maximum standardized uptake value (SUVmax), peak SUV (SUVpeak), total lesion glycolysis (TLG), and metabolic tumor volume (MTV), with histopathological and biochemical parameters in FDG-avid lung carcinoma, with a focus on tumor size, stage, and histological subtype.

Methods

This cross-sectional study included 45 patients diagnosed with FDG-avid lung carcinoma. FDG PET-CT imaging was performed to measure key biomarkers (SUVmax, SUVpeak, TLG, and MTV), and histopathological analysis was used to determine the tumor subtype. Biochemical markers, such as albumin, C-reactive protein (CRP), and neutrophil-to-lymphocyte ratio (NLR), were also evaluated. Correlation analyses were conducted using Pearson and Spearman tests, with multivariate regression models used to identify independent predictors of FDG uptake. Polynomial regression was employed to explore potential non-linear relationships between tumor size and FDG PET biomarkers.

Results

SUVmax showed a significant positive correlation with tumor size across all stages (r = 0.68, p < 0.001), with stronger associations in advanced stages (Stage II: r = 0.75, p = 0.02; Stage III: r = 0.80, p = 0.01). A strong correlation was found between TLG and MTV (r = 0.89, p < 0.001). Histological analysis revealed significantly higher SUVmax values in squamous cell carcinoma (17.4 ± 8.1) compared to adenocarcinoma (12.8 ± 6.4) (p = 0.04). Biochemical markers, including CRP and NLR, showed moderate correlations with PET biomarkers, particularly with TLG (r = 0.34, p = 0.03). Multivariate analysis revealed that tumor size and stage were significant predictors of SUVmax and TLG. Polynomial regression suggested a non-linear relationship between tumor size and FDG uptake, particularly in larger tumors.

Conclusion

FDG PET biomarkers, especially SUVmax, TLG, and MTV, provide valuable prognostic information in lung carcinoma, with stronger correlations observed in advanced tumor stages. Squamous cell carcinoma exhibited higher FDG uptake compared to adenocarcinoma. The integration of these biomarkers with histopathological and biochemical parameters can improve diagnostic accuracy and prognostication in lung cancer patients. Further studies are needed to explore the combined use of metabolic and systemic biomarkers to enhance treatment planning.

## Introduction

Lung carcinoma, one of the leading causes of cancer-related deaths globally, presents significant diagnostic and therapeutic challenges. Fluorodeoxyglucose positron emission tomography-computed tomography (FDG PET-CT) is a widely utilized non-invasive imaging modality that helps assess metabolic activity and tumor burden in lung cancer patients. Key FDG PET biomarkers, including metabolic tumor volume (MTV), total lesion glycolysis (TLG), maximum standardized uptake value (SUVmax), and peak SUV (SUVpeak), have shown potential for evaluating tumor aggressiveness and predicting patient outcomes [1]. These biomarkers offer an alternative to invasive tissue sampling, providing clinicians with crucial insights into tumor biology, particularly in cases where histopathological analysis is challenging [2].

Several studies have demonstrated that FDG PET-CT parameters, such as SUVmax, are correlated with tumor size, histological subtype, and molecular markers such as Ki-67 and EGFR mutations [3]. Despite these promising associations, the integration of FDG PET biomarkers with biochemical markers, such as albumin and lymphocyte count, remains underexplored. These biochemical parameters are known to reflect systemic inflammation and nutritional status, which may influence tumor metabolism [4]. The present study aims to assess the correlations between baseline FDG PET biomarkers and both histopathological and biochemical parameters in patients with FDG-avid lung carcinoma, contributing to the broader understanding of tumor behavior and potentially improving diagnostic and prognostic strategies.

This study was previously presented as a conference abstract at the National Conference on Recent Development in Medical Sciences (NeuroQuantology-Oncology Affairs in India), held on January 19, 2026, in Delhi, India.

## Materials and methods

Study design and setting

This retrospective observational study was conducted at Yenepoya Medical College, Mangalore, India, in collaboration with the Departments of Nuclear Medicine, Pathology, and Biochemistry. The study aimed to evaluate the relationship between FDG PET-derived metabolic biomarkers and histopathological as well as biochemical parameters in patients with FDG-avid lung carcinoma.

Ethical considerations

The study was approved by the Institutional Ethics Committee (IEC) (Approval No: IEC/054/2025). Given the retrospective nature of the study, the requirement for informed consent was waived. All patient data were anonymized prior to analysis, and confidentiality was strictly maintained.

Study population

The study included adult patients (≥18 years) with newly diagnosed, treatment-naïve, FDG-avid lung carcinoma who underwent baseline FDG PET-CT imaging within four weeks of histopathological diagnosis. Only cases with confirmed histopathological diagnosis based on biopsy or surgical specimens were included.

Patients who underwent PET-CT for restaging, recurrence evaluation, or treatment response assessment were excluded. Cases with incomplete clinical, imaging, histopathological, or biochemical data were also excluded.

Data collection and tumor staging

Clinical, imaging, histopathological, and biochemical data were collected retrospectively from medical records. Demographic variables included age and gender. Imaging-derived parameters included FDG PET biomarkers such as SUVmax, SUVpeak, MTV, and TLG.

Histopathological data included tumor subtype classification (adenocarcinoma, squamous cell carcinoma, or others), based on standard pathological criteria. Less common subtypes, including small cell carcinoma (if present), were grouped under the “other” category due to limited numbers.

Biochemical parameters included serum albumin, lactate dehydrogenase (LDH), C-reactive protein (CRP), and neutrophil-to-lymphocyte ratio (NLR). Tumor staging was performed using the Tumor-Node-Metastasis (TNM) classification according to the American Joint Committee on Cancer (AJCC), 8th edition, based on imaging and histopathological findings [5].

Imaging protocol and PET biomarker assessment

All patients underwent FDG PET-CT imaging for initial staging prior to initiation of treatment, following institutional protocols. Patients were instructed to fast for at least six hours prior to tracer administration, and blood glucose levels were confirmed to be within acceptable limits before imaging.

After intravenous administration of FDG, image acquisition was performed using a dedicated PET-CT scanner. Images were reconstructed using standard algorithms as per institutional protocol.

Regions of interest (ROIs) were delineated over the primary tumor using vendor-provided software (syngo.via, Siemens Healthineers, Erlangen, Germany) with semi-automated segmentation techniques. PET-derived metrics, including SUVmax, SUVpeak, MTV, and TLG, were calculated using standard software algorithms. All analyses were performed using a patient-based approach, with one primary FDG-avid lesion evaluated per patient.

Statistical analysis

All statistical analyses were performed using IBM SPSS Statistics for Windows, Version 25.0 (IBM Corp., Armonk, NY, USA). Continuous variables were expressed as mean ± standard deviation, while categorical variables were presented as frequencies and percentages.

Correlation analyses between FDG PET biomarkers and tumor size, stage, histopathological subtype, and biochemical parameters were performed using the Pearson correlation test or Spearman rank correlation, depending on data distribution. The direction of correlation was determined by the sign of the correlation coefficient (r), while the strength was classified based on its absolute value as follows: negligible (<0.20), weak (0.20-0.39), moderate (0.40-0.59), strong (0.60-0.79), and very strong (≥0.80). A p-value of <0.05 was considered statistically significant.

Multivariate regression analysis was performed to identify independent predictors of FDG uptake, particularly SUVmax and TLG. Variables such as tumor size, stage, and histopathological subtype were included in the model. Polynomial regression analysis was conducted to explore potential non-linear relationships between tumor size and PET biomarkers.

## Results

Study population and baseline characteristics

A total of 45 patients with newly diagnosed, treatment-naïve FDG-avid lung carcinoma were included in the study. The cohort comprised 26 men (57.8%) and 19 women (42.2%), with a mean age of 68.3 ± 7.4 years.

Histopathological evaluation revealed adenocarcinoma in 24 patients (53.3%), squamous cell carcinoma in 15 patients (33.3%), and other subtypes in six patients (13.3%). The “other” category included less common histologies, including small cell carcinoma, where present.

Based on TNM staging (AJCC 8th edition), 17 patients (38%) were classified as Stage I, 19 patients (42%) as Stage II, and nine patients (20%) as Stage III. The baseline characteristics and distribution of FDG PET biomarkers are summarized in Table 1.

**Table 1:** Baseline characteristics and distribution of FDG PET biomarkers in the study population Values are presented as mean ± standard deviation or frequency (%). Tumor staging was performed according to the Tumor-Node-Metastasis (TNM) classification based on the American Joint Committee on Cancer (AJCC), 8th edition [5]. FDG PET: fluorodeoxyglucose positron emission tomography; SUVmax: maximum standardized uptake value; MTV: metabolic tumor volume; TLG: total lesion glycolysis

Characteristic	Total (n = 45)	Adenocarcinoma (n = 24)	Squamous cell carcinoma (n = 15)	Other (n = 6)
Male	26 (57.8%)	14 (58.3%)	10 (66.7%)	2 (33.3%)
Female	19 (42.2%)	10 (41.7%)	5 (33.3%)	4 (66.7%)
Age (years)	68.3 ± 7.4	68.1 ± 7.3	68.6 ± 7.7	69.0 ± 7.1
Stage I	17 (38%)	9 (37.5%)	7 (46.7%)	1 (16.7%)
Stage II	19 (42%)	12 (50.0%)	6 (40.0%)	1 (16.7%)
Stage III	9 (20%)	3 (12.5%)	2 (13.3%)	4 (66.7%)
SUVmax	14.9 ± 7.2	12.8 ± 6.4	17.4 ± 8.1	15.6 ± 7.0
MTV (cm³)	42.5 ± 18.3	38.2 ± 15.6	48.7 ± 20.1	44.3 ± 17.8
TLG (g)	312.6 ± 145.2	280.4 ± 130.7	356.8 ± 160.3	330.5 ± 142.6
Histopathology features	-	Glandular differentiation, mucin production, TTF-1 positive	Keratinization, intercellular bridges, p40 positive	Includes small cell carcinoma/others

Correlation between FDG PET biomarkers and tumor characteristics

**Figure 1:**
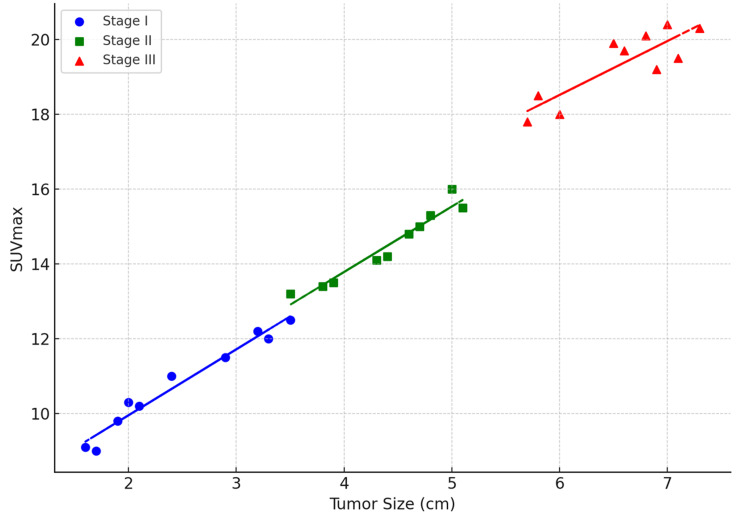
Scatter plot of SUVmax versus tumor size for each tumor stage Illustration of the correlation between tumor size and SUVmax across different tumor stages (Stage I, Stage II, and Stage III). Each stage is represented with different markers and colors. Additionally, trendlines are added for each stage to show the linear relationship between tumor size and SUVmax. SUVmax: maximum standardized uptake value

SUVmax demonstrated a strong, significant positive correlation with tumor size (r = 0.68, p < 0.001). When stratified by tumor stage, this relationship was moderate in Stage I (r = 0.52, p = 0.04), strong in Stage II (r = 0.75, p = 0.02), and strong in Stage III (r = 0.80, p = 0.01). This relationship is illustrated in Figure 1.

A very strong, significant positive correlation was observed between TLG and MTV (r = 0.89, p < 0.001). Stage-wise analysis showed moderate correlation in Stage I (r = 0.65, p = 0.03) and very strong correlations in Stage II and Stage III (r ≥ 0.90, p < 0.001). SUVpeak demonstrated a weak and non-significant correlation with tumor size (r = 0.09, p = 0.45).

Pearson correlation analysis further demonstrated strong, significant positive correlations of SUVmax with TLG (r = 0.71, p < 0.001) and MTV (r = 0.77, p < 0.001). These findings are summarized in Table 2.

**Table 2:** Pearson correlation coefficients (r) between FDG PET biomarkers, tumor size, and tumor stage Values represent Pearson correlation coefficients (r). Corresponding p-values are shown in parentheses. Statistical significance was defined as p < 0.05. FDG PET: fluorodeoxyglucose positron emission tomography; SUVmax: maximum standardized uptake value; SUVpeak: peak standardized uptake value; MTV: metabolic tumor volume; TLG: total lesion glycolysis

Variable	SUVmax	SUVpeak	TLG	MTV
Tumor size	0.68 (p < 0.001)	0.09 (p = 0.45)	0.60 (p < 0.001)	0.62 (p < 0.001)
Stage	0.61 (p < 0.001)	0.15 (p = 0.32)	0.58 (p < 0.001)	0.57 (p < 0.001)
SUVmax	1.00	0.09 (p = 0.45)	0.71 (p < 0.001)	0.77 (p < 0.001)
SUVpeak	0.09 (p = 0.45)	1.00	0.20 (p = 0.18)	0.12 (p = 0.34)
TLG	0.71 (p < 0.001)	0.20 (p = 0.18)	1.00	0.89 (p < 0.001)
MTV	0.77 (p < 0.001)	0.12 (p = 0.34)	0.89 (p < 0.001)	1.00

Histological subtype analysis

Comparison of metabolic parameters between histological subtypes revealed significantly higher FDG uptake in squamous cell carcinoma compared to adenocarcinoma. The mean SUVmax for squamous cell carcinoma was 17.4 ± 8.1, whereas adenocarcinoma demonstrated a mean SUVmax of 12.8 ± 6.4 (independent t-test, p = 0.04).

**Figure 2:**
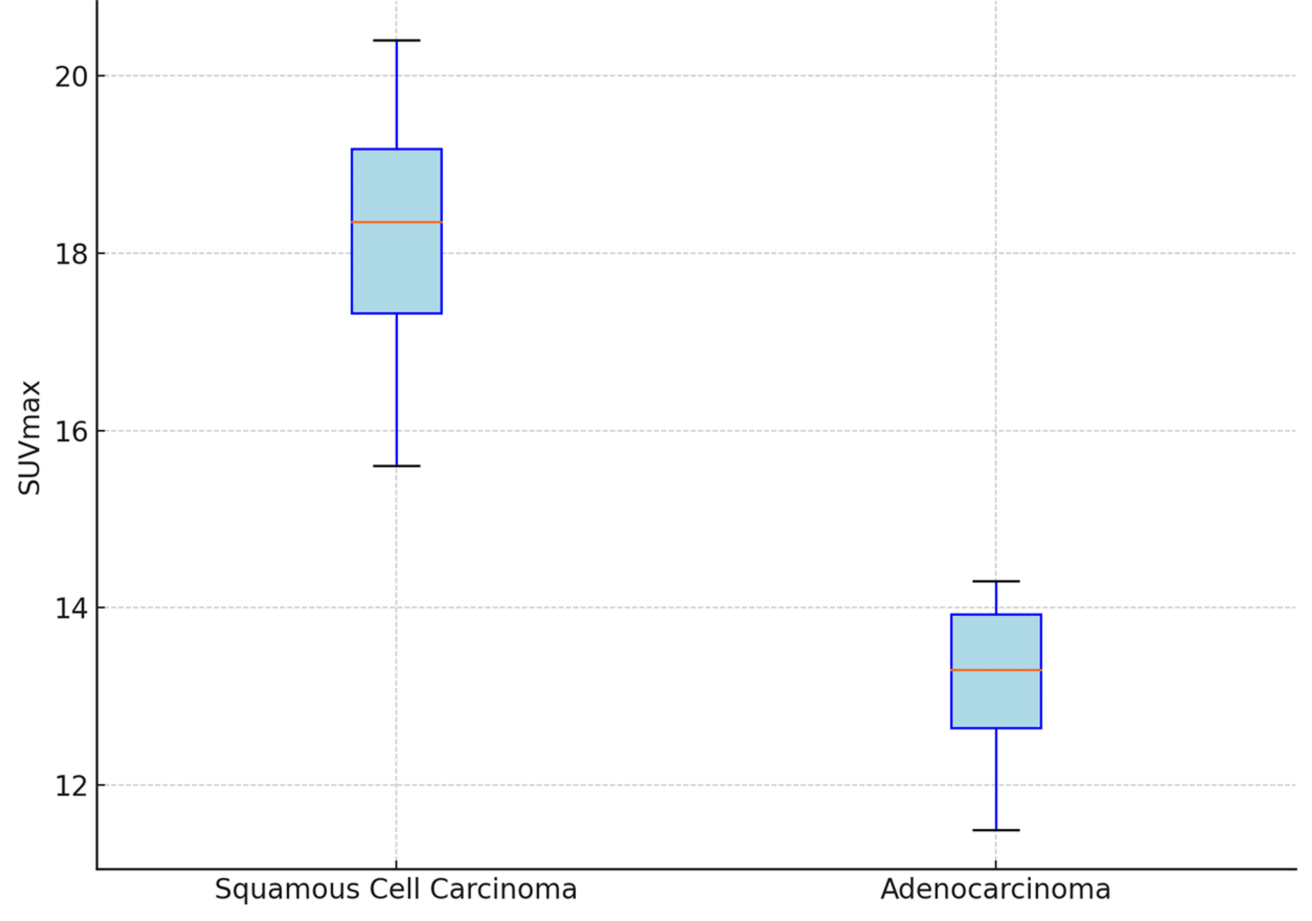
SUVmax across histological subtypes The figure shows the distribution of SUVmax across two histological subtypes: squamous cell carcinoma (SCC) and adenocarcinoma (ADC). The boxplot provides a clear comparison of the range and central tendency of SUVmax values for both subtypes, with noticeably higher SUVmax values in SCC compared to ADC. SUVmax: maximum standardized uptake value

The distribution of SUVmax across histological subtypes is illustrated in Figure 2, which demonstrates a higher median and spread of values in squamous cell carcinoma. Similarly, TLG and MTV values were significantly higher in squamous cell carcinoma compared to adenocarcinoma, with p-values of 0.03 and 0.02, respectively, indicating a higher metabolic tumor burden.

Correlation between FDG PET biomarkers and biochemical parameters

A moderate positive correlation was observed between CRP and TLG (r = 0.34, p = 0.03), suggesting an association between systemic inflammation and tumor metabolic activity. An inverse correlation was identified between NLR and SUVpeak (r = -0.45, p = 0.01), indicating that higher NLR values are associated with lower SUVpeak measurements. No statistically significant correlations were observed between albumin or LDH levels and FDG PET biomarkers.

Non-linear relationship between tumor size and PET biomarkers

Polynomial regression analysis demonstrated a statistically significant non-linear relationship between tumor size and FDG PET biomarkers. A quadratic association was observed for SUVmax (p = 0.03) and TLG (p = 0.02), indicating that metabolic activity increases disproportionately in larger tumors.

Multivariate analysis

Multivariate regression analysis identified tumor size and tumor stage as independent predictors of FDG uptake. Tumor size (β = 0.53, p = 0.001) and tumor stage (β = 0.42, p = 0.01) were significant predictors of SUVmax. Similarly, tumor size (β = 0.61, p < 0.001) and tumor stage (β = 0.37, p = 0.02) were independent predictors of TLG.

Subgroup analysis

In Stage I disease, SUVmax showed a moderate correlation with tumor size (r = 0.52, p = 0.04), while TLG (r = 0.45, p = 0.06) and MTV (r = 0.49, p = 0.05) demonstrated weaker associations. In contrast, in Stage II and Stage III disease, SUVmax showed stronger correlations with tumor size (Stage II: r = 0.75, p = 0.02; Stage III: r = 0.80, p = 0.01), while TLG and MTV demonstrated very strong correlations (r ≥ 0.90, p < 0.001), highlighting their reliability as indicators of tumor burden in advanced stages.

## Discussion

This study has several notable strengths. It evaluates a homogeneous cohort of treatment-naïve patients, minimizing the confounding effects of prior therapy on metabolic activity. Additionally, it integrates FDG PET-derived biomarkers with histopathological and biochemical parameters, allowing a multidimensional assessment of tumor behavior. The inclusion of volumetric PET parameters (MTV and TLG) alongside conventional SUV-based metrics provides a more comprehensive evaluation of tumor burden. Furthermore, the use of a patient-based analytical approach enhances clinical applicability and reflects real-world oncological decision-making.

The present study demonstrates that FDG PET-derived biomarkers, particularly SUVmax, MTV, and TLG, show significant correlations with tumor size and stage, supporting their established role in non-invasive tumor assessment [1-3]. The observed strong, significant positive correlation between SUVmax and tumor size is consistent with previous studies linking increased FDG uptake to tumor proliferation, hypoxia, and enhanced glycolytic metabolism [4,6]. The progressive strengthening of this correlation across tumor stages further suggests that metabolic activity increases with tumor advancement [7].

Volumetric PET parameters, especially MTV and TLG, showed stronger associations with tumor burden compared to SUVmax. This finding aligns with prior evidence indicating that volumetric parameters provide a more accurate representation of total tumor metabolic activity, as they incorporate both lesion size and metabolic intensity [8,9]. The very strong correlation observed between MTV and TLG further supports their interdependence and highlights their utility in comprehensive tumor evaluation [10].

Histological subtype analysis revealed significantly higher metabolic activity in squamous cell carcinoma compared to adenocarcinoma. This observation is in agreement with earlier reports suggesting that squamous tumors exhibit higher FDG uptake due to increased expression of glycolytic enzymes and higher proliferative activity [11,12]. However, overlap between subtypes was noted, indicating that PET biomarkers alone may not reliably differentiate histological subtypes without pathological correlation [6].

The moderate correlation between CRP and TLG observed in this study suggests a potential interaction between systemic inflammation and tumor metabolism. Inflammatory pathways are known to influence tumor progression and metabolic activity, which may explain the observed association [13,14]. However, biochemical correlations were less consistent overall, indicating that these relationships require further investigation.

Importantly, this study identified a non-linear relationship between tumor size and metabolic activity, suggesting that metabolic changes may not increase proportionally with tumor growth. This may reflect underlying tumor microenvironmental factors such as hypoxia, necrosis, and heterogeneous cellular composition in larger tumors [6,15].

Multivariate analysis confirmed that tumor size and stage are independent predictors of FDG uptake, reinforcing the concept that tumor burden is a primary determinant of metabolic activity [1,7]. These findings support the biological validity of FDG PET biomarkers as indicators of disease extent.

These findings are consistent with prior literature demonstrating the diagnostic and predictive utility of FDG PET-derived biomarkers in solid malignancies. Studies have shown that parameters such as SUVmax, MTV, and TLG serve as reliable indicators of tumor metabolic activity and disease burden, with volumetric parameters offering superior representation of whole-tumor behavior compared to single-voxel measurements [16,17].

Despite these strengths, several limitations must be acknowledged. The relatively small sample size limits statistical power, particularly for subgroup analyses. The retrospective and single-center design may introduce selection bias and limit generalizability. Although methodological details have been clarified, variations in imaging protocols across institutions may affect reproducibility. Additionally, the absence of survival analysis restricts interpretation to a correlative framework, and no prognostic conclusions can be drawn. Histopathological heterogeneity, including grouping of less common subtypes such as small cell carcinoma, may also mask subtype-specific biological differences [12,18,19].

Overall, this study supports the role of FDG PET-derived biomarkers, particularly volumetric parameters, as reliable non-invasive indicators of tumor burden and metabolic activity in lung carcinoma. However, further multicenter, prospective studies with larger cohorts are required to validate these findings and explore their potential clinical applications.

## Conclusions

This study demonstrates that FDG PET-derived metabolic biomarkers, particularly MTV and TLG, are robust indicators of tumor burden in FDG-avid lung carcinoma. These volumetric parameters showed strong and consistent correlations with tumor size and stage, outperforming single-voxel metrics such as SUVmax and SUVpeak in reflecting overall metabolic activity. SUVmax remains a useful and widely accessible parameter, with significant association with tumor size and histological subtype, particularly showing higher values in squamous cell carcinoma compared to adenocarcinoma. However, its limited representation of total tumor burden reduces its standalone prognostic utility.

The observed correlations between PET biomarkers and systemic inflammatory markers, especially CRP, suggest a potential link between tumor metabolism and host inflammatory response. Although these associations were modest, they indicate the value of integrating metabolic imaging with biochemical parameters for a more comprehensive assessment of tumor biology. The identification of a non-linear relationship between tumor size and FDG uptake further highlights the complexity of tumor metabolism, particularly in larger and more advanced lesions. This finding warrants further investigation in larger cohorts. Overall, the integration of FDG PET biomarkers-especially volumetric indices-with histopathological and biochemical parameters may enhance diagnostic accuracy, risk stratification, and individualized treatment planning in lung carcinoma. Future prospective studies with larger sample sizes and survival outcomes are required to validate these findings and establish their prognostic significance.
